# A Case of Calculous Concretions in the Tonsils

**Published:** 1800-05

**Authors:** John Hewitt

**Affiliations:** Surgeon, at Hull


### *A Caſe of calculous Concretions in the Tonſils.*

### *By* JOHN HEWITT, *Surgeon, at Hull.*

### *Communicated by Dr.* CLARKE.

MRS. H——, aged 39, and of a corpulent habit, about nine years ago, by expoſure to cold, was attacked with the uſual ſymptoms of inflamed ſore throat. The left tonſil was particularly inflamed, and came to ſuppuration. In about three months the abſceſs broke, and diſcharged matter of proper conſiſtence. Three days afterwards, the ſurgeon who then attended her, introduced a probe, and very diſtinctly felt a hard, rough ſubſtance. On the ſame day, a ſtone, of a light yellow colour, and about the ſize of a ſmall field bean, came from the orifice. She had at this time a child at the breaſt, eighteen months old. She remained a widow five years; in her ſixth year ſhe was married to her ſecond huſband, and again became pregnant. I attended her during her pregnancy, when ſhe parted with two concretions from the ſame tonſil, both ſmaller than the firſt; but without any previous inflammation or ſuppuration. She again became pregnant, and parted with a ſtone as the laſt. About the month of July, 1799, during her laſt pregnancy, the glands of the neck were much ſwollen, but ſhe felt no pain, nor was there any appearance of inflammation. One month, however, after delivery, the left tonſil gland became very much inflamed, and in a fortnight ſuppurated. The diſcharge was conſiderable for a few days; after which I introduced my finger into her mouth, and felt the end of the ſtone; by gentle preſſure forwards, it came into her mouth. It weighed 11 grains, and was exactly of the form and colour that I ſent it. I ſhould have tried two or three chemical experiments upon it, but I wiſhed you to ſee it in its perfect form.

